# The key role of muscle spindles in the pathogenesis of myofascial trigger points according to ramp-and-hold stretch and drug intervention in a rat model

**DOI:** 10.3389/fphys.2024.1353407

**Published:** 2024-05-14

**Authors:** Lin Liu, Shi-Xuan Liu, Qiangmin Huang, Qing-Guang Liu

**Affiliations:** ^1^ Department of Rehabilitation, School of Sport Health, Nanjing Sport Institute, Nanjing, China; ^2^ Department of Sport Medicine and the Center of Rehabilitation, School of Sport Science, Shanghai University of Sport, Shanghai, China; ^3^ Department of Pain Medicine, Shanghai Yiyang TCM Clinic, Shanghai, China; ^4^ International College of Football, Tongji University, Shanghai, China

**Keywords:** muscle spindles, trigger points, reflex, stretch, succinylcholine, eperisone

## Abstract

This study investigated whether abnormal peak inversion spontaneous potentials (PISPs) recorded at resting myofascial trigger points (MTrPs) stem from the discharge of muscle spindles. Forty-eight male Sprague-Dawley rats were randomly divided into six groups. Five groups underwent MTrP modeling intervention, whereas one group did not receive intervention and was duly designated as the blank control. After model construction, five rat models were randomly subjected to ramp-and-hold stretch tests, succinylcholine injection, eperisone hydrochloride injection, saline injection, and blank drug intervention. By contrast, the rats in the blank control group were subjected to ramp-and-hold stretch tests as a control. Frequencies and amplitudes of PISPs were recorded pre- and post-interventions and compared with those of the blank group. Stretch tests showed that the depolarization time and amplitude of PISPs ranged from 0.4 ms to 0.9 ms and from 80 uV to 140 μV, respectively. However, no PISPs were observed in the control rats. The frequency of PISPs in the ramp and hold phases and the first second after the hold phase was higher than that before stretching (*p* < 0.01). Succinylcholine and eperisone exerted excitatory and inhibitory effects on PISPs, respectively. In the group injected with 0.9% saline, no considerable differences of the PISPs were observed during the entire observation period. In conclusion, PISPs recorded at resting MTrPs are closely related to muscle spindles. The formation of MTrPs may be an important factor that regulate dysfunctional muscle spindles.

## 1 Introduction

Myofascial trigger points (MTrPs) are among the most common causes of enigmatic musculoskeletal pain and dysfunction. Researchers and physicians are now paying increased attention to MTrPs because of their high prevalence (Sánchez-Romero et al., 2019; Sánchez Romero et al., 2020). MTrPs are some hyperirritable spots in taut bands of skeletal muscles. MTrPs exhibit the dual pathophysiological features of cell morphology and spontaneous discharge of muscles (Huang et al., 2015; Zhang et al., 2017). MTrPs can cause soft tissue pain of body and interfere with adjacent blood vessels, nerves, fascia, and other structures, inducing vascular diseases, neurological diseases, pelvic floor dysfunction, menstrual cycle disorders, cardiovascular diseases, and sports injuries (Simons, 2003; Huang and Liu, 2014; Zhuang et al., 2014; Tang et al., 2018; Huang and Zhang, 2019). Previous studies on MTrP pathogenesis suggested that abnormal motor endplate functions in skeletal muscles lead to excessive release of acetylcholine at the endplate, resulting in persistent muscle contracture and chronic MTrPs (Liu et al., 2019; Martínez-Jiménez et al., 2022; San-Antolín-Gil et al., 2022). Hubbard and Berkoff (Hubbard and Berkoff, 1993) recorded the peak potential at MTrPs by using unipolar electromyographic needles. They postulated that the discharge activity might originate from abnormal muscle spindles in skeletal muscles.

Muscle spindles are some important proprioceptors that provide information on muscle length, including the rate of change in muscle length in skeletal muscles (Capra and Ro, 2000) They play an important role in maintaining muscular tension and fine modulation of voluntary movements (Johansson and Sojka, 1991) Previous studies claimed that the formation of myofascial pain syndrome affected the function of muscle spindles, especially in skeletal muscles with high-density muscle spindles (Johansson and Sojka, 1991; Matre et al., 1998; Liu et al., 2003). A prior study that employed electromyography (EMG) demonstrated that several peak inversion spontaneous potentials (PISPs) can be recorded in MTrPs at rest, and these PISPs are vastly different from the endplate potentials of normal skeletal muscles (Huang et al., 2013; Huang et al., 2015) We also suspected that these abnormal PISPs may originate from the discharges of two kinds of endings, namely, primary and secondary endings, innervating muscle spindles. Prior to the present study, no conclusive experimental evidence has been presented to prove this hypothesis. In order to investigate whether abnormal PISPs were related to muscle spindles, we planned to adjust the changes of muscle spindles through various intervention methods to observe the changes of abnormal PISPs after muscle spindles were regulated.

The primary and secondary endings of muscle spindles in animals are supplied by Ia and II fibers, respectively. These fibers exhibit different responses to imposed ramp-and-hold stretch (De-Doncker et al., 2003). Discharge of Ia fibers indicate both muscular length changes (static sensitivity) and velocity of length changes (dynamic sensitivity) (McCloskey, 1978). By comparison, discharge of II fibers chiefly provide information on length changes (McCloskey, 1978; De-Doncker et al., 2003). The extent by which sensory endings are innervated in intrafusal fibers reflects a capacity for depolarization of sensory endings because the stretch of intrafusal fibers mechanically deforms the sensory terminals, thereby altering the ionic permeability of the sensory terminals, a process that in turn depolarizes the terminal and causes muscle spindle discharge (Kim et al., 2007). Therefore, the primary purpose of the present study was to investigate the effects of ramp-and-hold stretch on the discharge of abnormal PISPs recorded at resting MTrPs to determine the relationship between the abnormal PISPs of MTrPs and spindle discharges.

Succinylcholine (SCh) can increase the afferent discharge of muscle spindles, which is often used to excite muscle spindles in laboratory (Granit et al., 1953; Rack and Westbury, 1966). Its mechanism of exciting muscle spindles is similar to that of stimulating motor nerve of dynamic muscle spindles, which can excite intrafusal fibers and make them contract at both extremes, simulating the action of γ-motoneurons (Rack and Westbury, 1966). Furthermore, SCh has strong muscle selectivity, quick onset, short duration, and allows full recovery. Hence, SCh can be used as a drug for experimentally exciting the spontaneous electrical activity of muscle spindles. Eperisone hydrochloride is a nondepolarizing muscle relaxant. It can suppress the spontaneous discharge of γ-motoneurons in animals, thereby reducing the sensitivity of muscle spindles (Vasavi and Perumal, 2019). In this study, SCh and eperisone hydrochloride were respectively injected into MTrPs to observe changes in abnormal PISPs in spontaneous EMG of the MTrPs. Our ultimate aim was to determine the effect of MTrPs formation on muscle spindles.

## 2 Materials and methods

### 2.1 Experimental animals

Forty-eight specific pathogen-free (SPF) male Sprague–Dawley rats (7 weeks, 220–240 g) were obtained commercially from Shanghai Laboratory Animal Center (Shanghai, China, animal License No. SCXK 2007-0003). They were housed in a pathogen-free animal facility maintained at 20°C–22°C, 45%–55% RH, and 12 h/12 h light/dark cycle with *ad libitum* access to food and water and minimal environmental noise. After 1 week of environmental adaptation, animals were randomly assigned to five groups (*n* = 8 for each group): four MTrP model groups, namely, Group M1, Group M2, Group M3, and Group M4 (*n* = 32), as well as a non-MTrP normal group (Group N1, n = 8). Guidelines of the National Institutes of Health Guide for the Care and Use of Laboratory Animals were followed for all experiments. All possible efforts were made to minimize the number of experimental animals used and reduce their suffering. The Committee on the Ethics of Animal Experiments of Shanghai University of Sport (Approval No. 2014012) granted ethical approval for animal use.

### 2.2 Experimental protocol

Modeling Intervention: As described in our previously reported method (Huang et al., 2013; Huang et al., 2015; Zhang et al., 2017; Liu et al., 2019), a MTrP model was established by blunt striking on the gastrocnemius muscle and eccentric-based exercise for 8 weeks along with 4 weeks of recovery. Briefly, the left proximal gastrocnemius muscle of rats in model groups was marked and struck by a homemade stick device dropped from a height of 20 cm with a kinetic energy of 2.352J to induce muscle contusion once every first day of each week. Then, rats were run on a treadmill (DSPT-202, Duanshi Co., Hangzhou, China) for 90 min at a −16° downward angle and speed of 16 m/min every second day.

When the rats showed corresponding characteristics of MTrPs described in the previous literature (Huang et al., 2013; Huang et al., 2015; Zhang et al., 2017; Liu et al., 2019), the model was considered to be constructed successfully. At first, a contracture nodule in a taut band was palpated at the hit site of the left gastrocnemius muscle. An electrode needle was then inserted into the contracture nodule. If a local twitch response (LTR) was observed to be elicited by needling, the contracture nodule was considered a possible MTrP. Second, for further confirmation, an EMG device three fine-needle electrodes (Φ0.3 mm) (NeuroCare-E, NCC Medical Co., Ltd., Shanghai, China, sampling frequency at 50,000 Hz) was used to record myoelectrical signals at the contracture nodule. When spontaneous electromyography activity (SEA) with a long duration (at least 120 s) of high frequency and amplitude (more than 130 µV) was detected, it was considered to represent a genuine active MTrP.

Ramp-and-hold Stretch Tests: The rats in Groups M1 and N1 were fixed on a platform in the supine position. A pulley was installed on the right side of the platform. The ankles of the rats were tied with thin wires, and then the wires were hung around the pulley to suspend the corresponding load. The recording electrode was placed at the MTrPs, whereas the other electrode was placed in the nearby muscle tissues (for the non-MTrP model group) as the reference electrode. Prior to the conduct of ramp-and-hold stretch tests, abnormal PISPs were obtained *via* EMG at the MTrPs of gastrocnemius muscles in a group of model rats. The initial length of muscle stretch (i.e., the initial length of rat gastrocnemius muscle under preload, L_0_) was set. The optimal stretch load of the ramp-and-hold stretch was determined by repeatedly stimulating gastrocnemius spindles by hanging different loads of weights (50, 100, 200, 300, 400, and 500 g). The range of stretch was 20% L_0_, the rate of stretch was maintained at 5 mm/s, the stable period of stretch was sustained at 3 s, and the interval was set to 45 s (Zhu et al., 2008). The spontaneous EMG of MTrPs and normal skeletal muscles under ramp-and-hold stretch were simultaneously recorded.

Drug Intervention: The rats in Groups M2, M3, and M4 were anesthetized with an injection of pentobarbital sodium. Afterward, the rats underwent tracheal intubation and external jugular vein intubation. Artificial respiration was performed on the rats at a frequency of 80 times per min with a tidal volume of 1.5–2.0 mL. Anal temperature was kept between 37°C and 38°C. The EMG of the rats was constantly monitored, whereas their pupils and skin color were observed any time to ensure that the rats were in a good functional state during the conduct of experiments. Active MTrPs were identified following a previously described method with a slight modification. The silver needle at recording electrode was replaced with a disposable 0.5 mm × 38 mm medical dental injection needle to ensure precise injection of normal saline and different drugs into the MTrPs. Before drug intervention was commenced, abnormal PISPs were first observed on the EMG of gastrocnemius MTrPs of the rats. The position of the electrodes were maintained. Subsequently, 100 mg/2 mL of medical-grade SCh (Shanghai Xudong Haipu Pharmaceutical Co.,Ltd, China) was injected into the active MTrPs located in the gastrocnemius muscles of the rats in Group M2 at a dose of 4 mg/kg. The frequency, depolarization time, and maximum amplitude of the abnormal PISPs at MTrPs were recorded within 1 min pre-injection and 30 s, 1–2 min, 2–3 min, and 3–5 min after administration. The rats in Group M3 were injected with 10% eperisone hydrochloride solution (Eisai China Inc, China) into the active MTrPs at a dose of 5 mg/kg. However, two injections of the same animal had no sufficient interval (>25 min). Similarly, the frequency, depolarization time, and maximum amplitude of abnormal PISPs at MTrPs were observed on the needle EMG 1 min pre-injection and 30 s, 1–2 min, 2–3 min, and 3–5 min after administration. The rats in Group M4 were selected as the drug control group. They were injected with normal saline at the same dose. The frequency, depolarization time, and maximum amplitude of abnormal PISPs at MTrPs were also observed on the needle EMG 1 min pre-injection and 30 s, 1–2 min, 2–3 min, and 3–5 min after administration.

### 2.3 Statistical analysis

Statistical processing of the obtained results and the reliability of differences were assessed by Student’s t-test. All statistical analyses were completed using PRISM version 5.01 (GraphPad Software, Inc., La Jolla, CA, USA). The level of significance of all the tests was set at *p* < 0.05. The results were presented as mean values and standard deviation (SD).

## 3 Results

### 3.1 Results of ramp-and-hold stretch intervention

**FIGURE 1:**
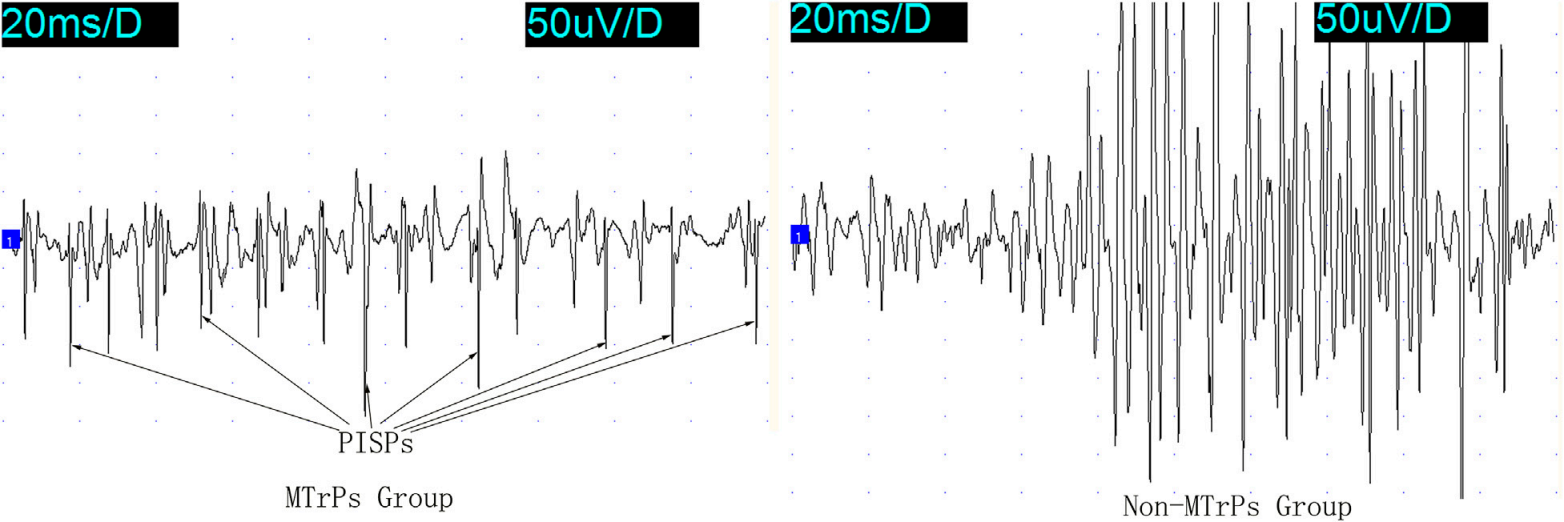
PISPs and Non-PISPs recorded at the MTrPs and Non-MTrPs group during single ramp-and-hold stretching test. Abbreviation: MTrPs, myofascial trigger points. PISPs, peak inversion spontaneous potentials.

**FIGURE 2:**
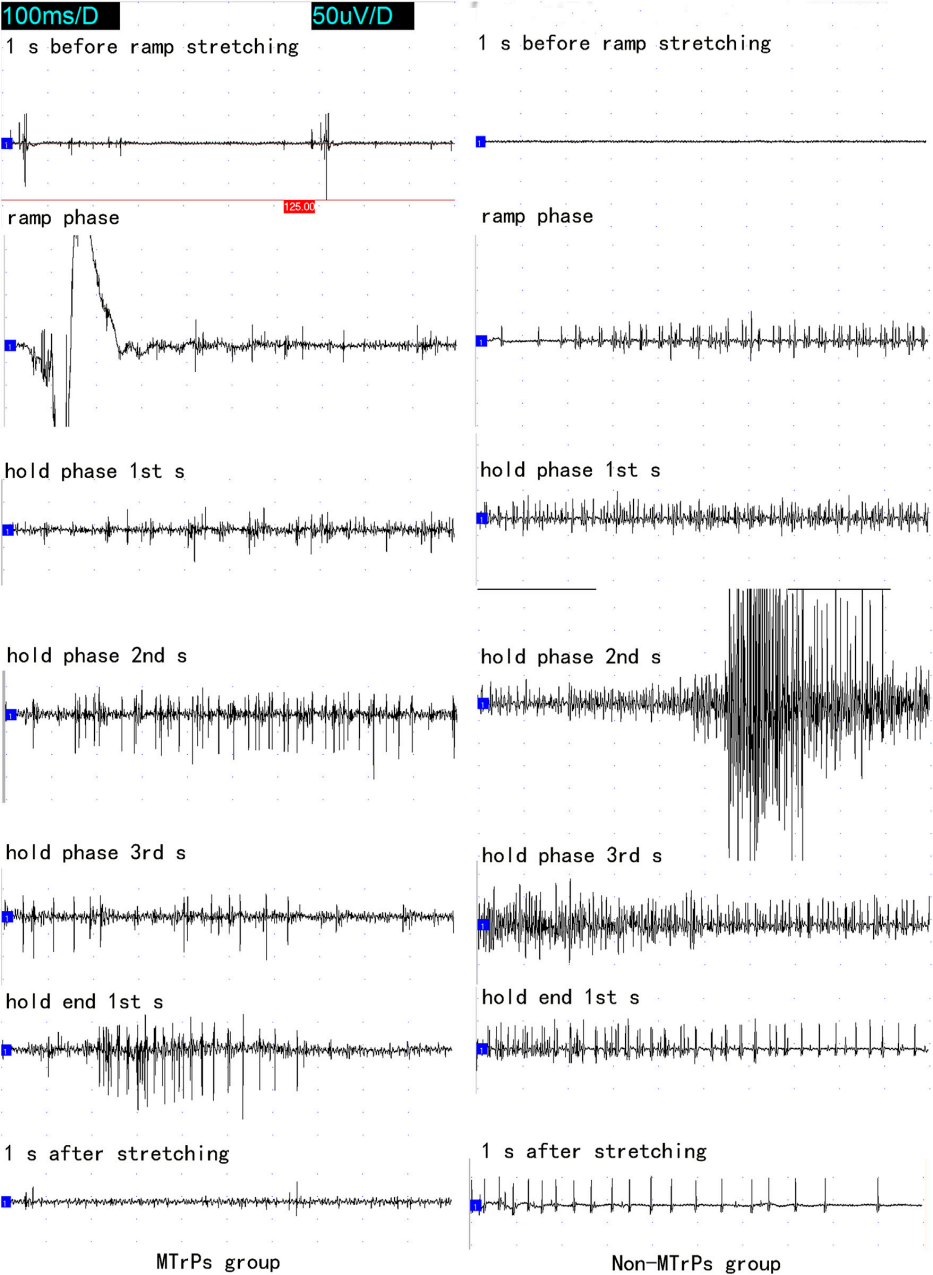
The whole course of needle electromyography recorded at the MTrPs and Non-MTrPs group during single ramp-and-hold stretching test. Abbreviation: MTrPs, myofascial trigger points.

Several ramp-and-hold pulling tests revealed that 400 g was the optimal pulling load. The effects of ramp-and-hold stretch on abnormal PISPs in MTrPs are shown in Figures 1, 2. Repeated ramp-and-hold stretching revealed that the depolarization time and amplitude of abnormal PISPs ranged from 0.4 ms to 0.9 ms and from 80 µV to 140 μV, respectively. However, no abnormal PISPs were observed during repeated ramp-and-hold stretching tests in normal control rats.

**FIGURE 3:**
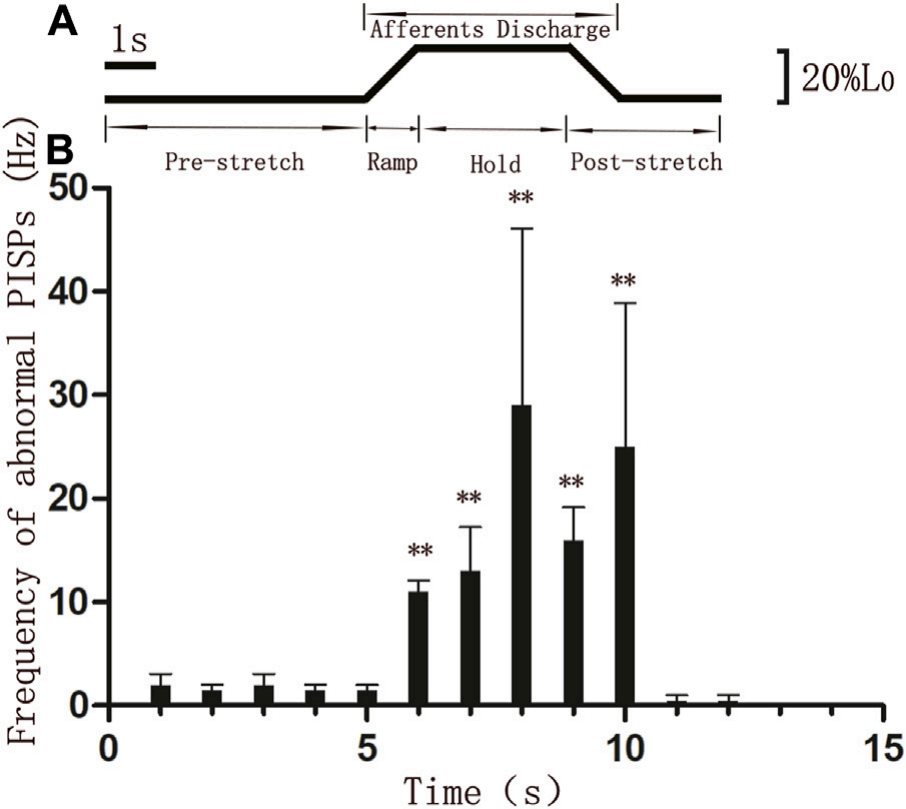
Discharging frequencies of abnormal PISPs during single ramp-and-hold stretching test **(A)** The length of MTrP rat gastrocnemius. L0, The initial length of rat gastrocnemius muscle under preload. **(B)** Discharging frequencies of abnormal PISPs before (0 s to 5th s), during (ramp phase: 6th s, hold phase: 7th s to 9th s) and after stretching. **p < 0.01 compared to 1 s before ramp stretching. Abbreviation: MTrP, myofascial trigger point. PISPs, peak inversion spontaneous potentials.

The discharging frequency of abnormal PISPs in ramp phase (11 ± 1.069 Hz, *n* = 8), hold phase 1st s (13 ± 4.276 Hz, *n* = 8), hold phase 2nd s (29 ± 17.105 Hz, *n* = 8), hold phase 3rd s (16 ± 3.207 Hz, *n* = 8), and hold end 1st s (25 ± 13.898 Hz, *n* = 8) were significantly higher than those in 1 s before ramp stretching (1.5 ± 0.535 Hz, *n* = 8) (*p* < 0.01, Figure 3). The highest discharge of abnormal PISPs was observed in hold phase 2nd s, and the frequency of abnormal PISPs gradually returned to the pre-stretch frequency after hold phase 2nd s.

In the Non-MTrPs group, the amplitude of electromyography did not show the greater excitement until the hold phase 2nd s. However, the greater excitement began at the ramp phase in the MTrPs group. It is thus clear that the excitation of the MTrPs group by stretching was earlier than that of the Non-MTrPs group.

### 3.2 Results of drug intervention

The intervention effects of SCh and eperisone on abnormal PISPs recorded at MTrPs are shown in Supplemental Figures S1–4. Compared with the blank control group, SCh significantly increased the amplitude of PISPs within 41–60 s post-injection (*p* < 0.01), and also continuously increased the frequency of PISPs within 4 min post-injection (*p* < 0.05, except for the two time periods of 11–20 s and 31–40 s). Additionally, eperisone continued to inhibit the potential amplitude and frequency of PISPs within 7 min post-injection (*p* < 0.01).

**FIGURE 4:**
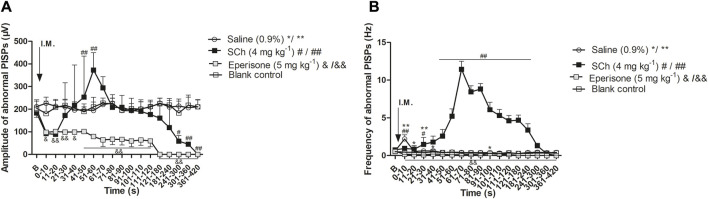
Effects of SCh and eperisone on discharging amplitude and frequencies of abnormal PISPs. **(A)** Effects of SCh and eperisone on discharging amplitude of abnormal PISPs, **(B)** Effects of SCh and eperisone on discharging frequencies of abnormal PISPs. *p < 0.05, **p < 0.001, #*p* < 0.05, ##*p* < 0.001, &*p* < 0.05 and &&*p* < 0.001 compared with the I.M. injection of blank control. B, baseline responses before the I.M. injection of 0.9% saline, SCh or eperisone. Abbreviation: SCh, succinylcholine. PISPs, peak inversion spontaneous potentials.

Based on the whole process after injection, the amplitude and frequency of PISPs after SCh injection initially decreased and then rapidly increased until they reached a peak within 51–70 s post-injection (Figure 4). The peak amplitude and frequency had a short-term stimulating effect on the induction of abnormal PISPs. The excitement lasted for about 1 min. However, the amplitude and frequency of spontaneous endplate potentials at MTrPs gradually decreased after SCh and substantially injection until them completely disappeared. The rats injected with saline and the rats in the model control group had no remarkable difference in amplitude and frequency of the PISPs during the entire observation period. Nevertheless, a transient increase in amplitude and frequency of the PISPs was immediately observed and 91–100 s after saline administration.

## 4 Discussion

The source of abnormal PISPs in spontaneous EMG of resting MTrPs has been puzzling us. The present study established that these abnormal PISPs of MTrPs are closely related to neighboring dysfunctional muscle spindles.

Muscle spindles are the main somatosensory receptors most sensitive to muscle stretch stimulation. When intrafusal fibers are stretched, the sensory endings of Ia afferents are stimulated and excited. The nerve response of spiral endings on nuclear bag fibers is dynamic, that is, the frequency of discharge substantially increases as muscle length increases) (Miwa et al., 1995; Schafer, 1995). Although excited contraction of nuclear chain fibers can considerably increase in II afferent impulses, the length sensitivity of II afferent fibers can remarkably increase when continuously stretched. Ramp-and-hold stretching of skeletal muscles can enhance spindle afferent signals to the spinal cord center *via* muscle elongation. The afferent signals can then improve the efferent signals of α-motorneurons *via* single synaptic processing of the spinal cord center. As a result, outgoing signals increase, leading to contractions of the extrafusal muscle fibers dominated by α-motorneurons. The tension of intrafusal muscle fibers eventually ends, and the discharge frequency of muscle spindle afferents decreases (Schafer, 1994; Schafer et al., 1999; De-Doncker et al., 2003). During ramp-and-hold stretching, nuclear bag fibers are mainly sensitive to stretching rates, whereas nuclear chain fibers are largely sensitive to length changes (De-Doncker et al., 2003). After subjecting MTrP skeletal muscles to ramp-and-hold stretch tests, the abnormal PISPs at MTrPs substantially increased during the ramp stretching period, whereas the discharge frequency notably increased during the entire hold period and the 1st s after the end of hold period. Therefore, the increase in the frequency of abnormal PISP discharges were both related to the stimulating effects of Iα and II afferents, that is, the generation of abnormal PISPs was related to the activation of nucleus chain fibers and nuclear bag fibers of muscle spindles.

Although ramp-and-hold stretching experiments alone cannot completely confirm that abnormal PISPs originate from muscle spindles, the discharge activity of muscle spindles can be regulated by various drugs, such as SCh and eperisone. SCh is a depolarizing muscle relaxant (it blocks the transmission of extrafusal nerve–muscle junction) and also a classical muscle spindle stimulant (Granit et al., 1953; Rack and Westbury, 1966). Nevertheless, the excitation effects of SCh can only be achieved within a certain concentration range (50–75 mg/L) (Matsunaga et al., 1997). When its concentration exceeds 100 mg/L, the excitement of muscle spindle afferents by SCh disappears and exerts inhibitory effects on muscle spindle afferents instead (Matsunaga et al., 1997). Shi et al. (Shi et al., 1996; Shi et al., 2003) reported that the peak discharge peak frequency of muscle spindle afferents in rats is the highest when SCh injection is 4 mg/kg, similar to that observed in the present study when SCh was intramuscularly injected into the MTrPs. Eperisone chiefly acts on the central nervous system by inhibiting the stretch reflex at the spinal cord level to reduce muscle tension, as well as by reducing the discharges of γ-motoneurons to suppress the sensitivity of muscle spindles (Matsunaga et al., 1997; Yang et al., 2004). On the basis of this principle and by referring to previously reported data, the present work selected 5 mg/kg of eperisone solution for intramuscular injection into the MTrPs in rats to observe whether PISPs can be inhibited (Matsunaga et al., 1997).

The results of the present study showed that the amplitude and frequency of PISPs briefly decreased and then rapidly increased until they reached a peak within 51–70 s after SCh administration into the MTrPs to interfere with abnormal PISPs. SCh stimulated Ia muscle spindle afferents to transiently excite by contracting intrafusal muscle fibers, eventually inducing a temporary excitement of the abnormal PISPs. The duration of this state of excitation was about 1 min. Careful EMG examination also revealed that the amplitude and frequency of spontaneous endplate potential gradually decreased with time until they completely disappeared, indicating that SCh inhibited the excessive release of acetylcholine at the neuromuscular junction. By contrast, the amplitude and frequency of PISPs and endplate potential both rapidly decreased after eperisone injection until they entirely disappeared, suggesting that eperisone injection reduced the induction of abnormal PISPs by inhibiting the activity of γ-motoneurons. However, opposite results were observed in the rats injected with saline. No remarkable differences in amplitude and frequency of the PISPs in the saline group were noted during the entire observation period, except for a transient increase in the amplitude and frequency of PISPs 91–100 s after administration. This result further proved that SCh and eperisone exerted excitatory and inhibitory effects on abnormal PISPs. Moreover, the results further suggested that the formation of abnormal PISPs may be closely related to abnormal discharge of intrafusal muscle fibers and hyperactivity of γ-motoneurons.

Simons et al. proposed that MTrPs are located at motor endplates of skeletal muscle fibers, and acupuncture of MTrPs can induce the classical endplate noise on EMG (Simons, 2004; Chou et al., 2009). Unlike their research, this study suggested that once MTrPs are formed, the spontaneous discharge of MTrPs may sensitize Iα and II afferents, thereby enhancing excitability of α- and γ-motoneurons through spinal central reflexes.The phenomenon of enhanced muscle spindle reflex caused by MTrPs is consistent with the viewpoint proposed by Matre et al. that experimental myalgia increases human stretch reflex (Matre et al., 1998; Capra and Ro, 2000). The excitability of motoneurons ultimately leads to excessive release of acetylcholine at motor endplates of both extrafusal and intrafusal muscle fibers. The excessive release of acetylcholine at motor endplates of extrafusal muscle fibres leads to the formation of endplate noise at MTrPs, while the release of acetylcholine at motor endplates of intrafusal muscle fibers aggravates the discharge of muscle spindles. In our study, not only endplate noise, but also high-frequency and high-amplitude PISPs unlike endplate noise can be observed during acupuncture at MTrPs, indicating that these PISPs may come from the discharge of sensitized muscle spindles. After further ramp-and-hold stretching and drug intervention on theses PISPs of these suspected muscle spindle discharges, theses PISPs showed the same electrophysiological characteristics as the discharge of muscle spindles. Recent histological examination results of MTrPs cells showed that there are abnormal muscle spindles among a group of MTrPs cells and MTrPs cells were much closer to the neighbouring muscle spindles anatomically (Liu et al., 2020). Based on the above evidence, we believe that it is highly likely that these suspected PISPs of the dischage of muscle spindles originate from muscle spindles. In clinical, this study reminded clinicians that the MTrPs formed in the patient’s body can disrupt the proprioceptive receptors of affected muscles (especially muscle spindles). Therefore, when treating chronic pain caused by the musculoskeletal system, clinicians should not only inactivate MTrPs, but also take necessary measures to regulate the function of muscle spindles, such as stretching (Martín-Pintado Zugasti et al., 2014; Budini et al., 2017).

## 5 Conclusion

The convincing electrophysiological evidence presented herein established that abnormal PISPs in spontaneous EMG of MTrPs is closely related to muscle spindles, that is, the formation of MTrP cells is closely related to the surrounding dysfunctional muscle spindles. This study opens a new door for exploring the relationship between muscle spindles and MTrPs.

## Data Availability

The raw data supporting the conclusion of this article will be made available by the authors, without undue reservation.

## Supplementary material

SUPPLEMENTARY FIGURE S1
Resting electromyography and abnormal PISPs at MTrPs before succinylcholine and eperisone injection. Notes: A and C, spontaneous EMG recorded within 10 s at a scanning speed of 200 ms before succinylcholine and eperisone injection. 5 EMGs in Figure A and Figure C are continuous records, and each EMG represents the spontaneous EMG changes within 2 s. (B,D), abnormal PISPs at MTrPs before succinylcholine and eperisone injection and they were selected from Figure A and Figure C, respectively. Abbreviation: MTrPs, myofascial trigger points. PISPs, peak inversion spontaneous potentials.

SUPPLEMENTARY FIGURE S2
Resting EMG recorded at MTrPs within 1 min after succinylcholine and eperisone injection. Notes: 5 EMGs in every 10 s are continuous recordings, and each EMG represents spontaneous EMG changes within 2 s. Abbreviation: MTrPs, myofascial trigger points. PISPs, peak inversion spontaneous potentials.

SUPPLEMENTARY FIGURE S3
Resting EMG recorded at MTrPs within 1–2 min after succinylcholine and eperisone injection. Notes: 5 EMGs in every 10 s are continuous recordings, and each EMG represents spontaneous EMG changes within 2 s. Abbreviation: MTrPs, myofascial trigger points. PISPs, peak inversion spontaneous potentials.

SUPPLEMENTARY FIGURE S4
Resting EMG recorded at MTrPs within 10 s at 3, 4, 5, 6, and 7 min after succinylcholine and eperisone injection. Notes: 5 EMGs in every 10 s are continuous recordings, and each EMG represents spontaneous EMG changes within 2 s. Abbreviation: MTrPs, myofascial trigger points. PISPs, peak inversion spontaneous potentials.
