# Supplementary figures and images for: The key role of muscle spindles in the pathogenesis of myofascial trigger points according to ramp-and-hold stretch and drug intervention in a rat model
Source: Front Physiol. 2024 May 14;15:1353407. doi: 10.3389/fphys.2024.1353407 (PMC11130495; doi:10.3389/fphys.2024.1353407)

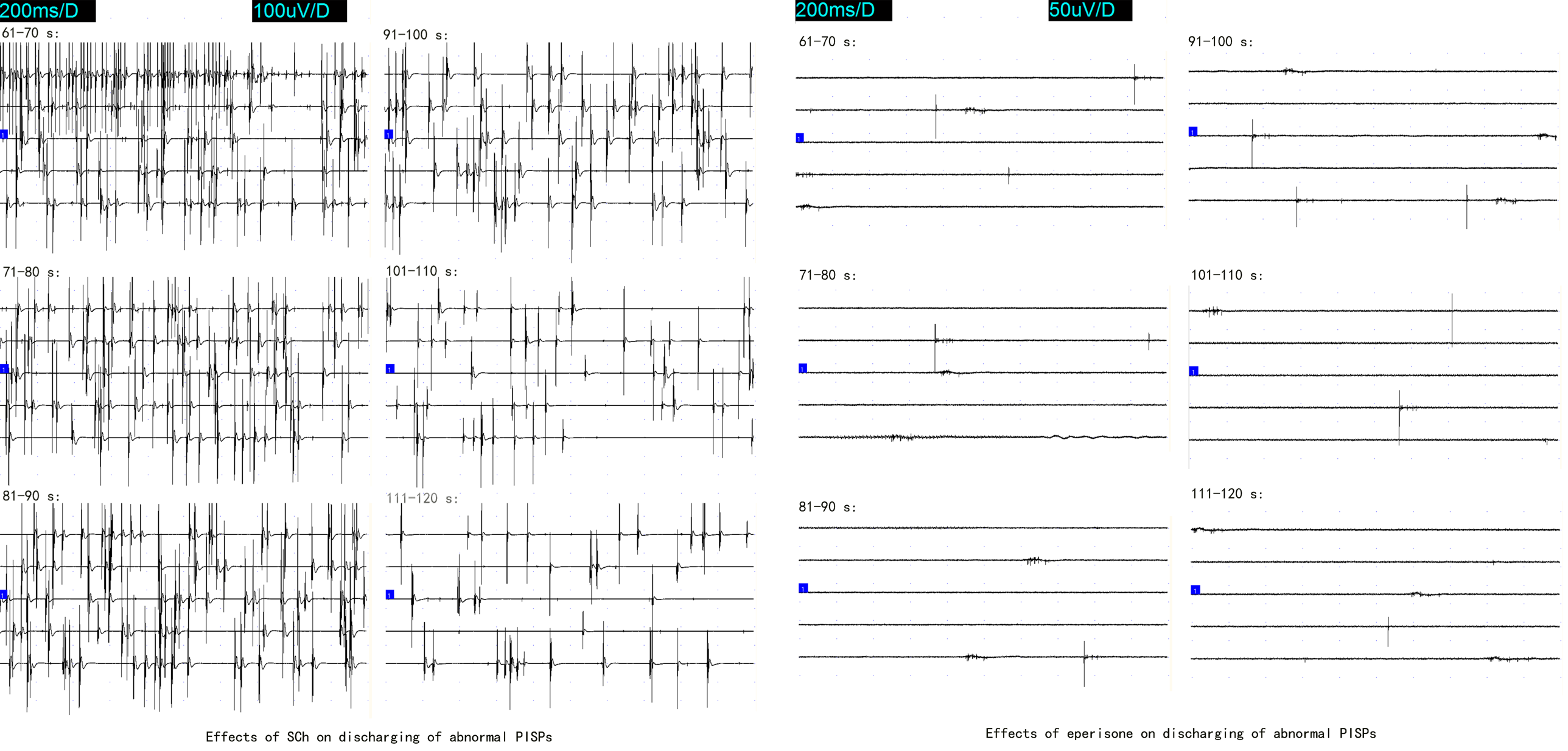

Supplement: Supplementary file 1 [file Image3.TIF]

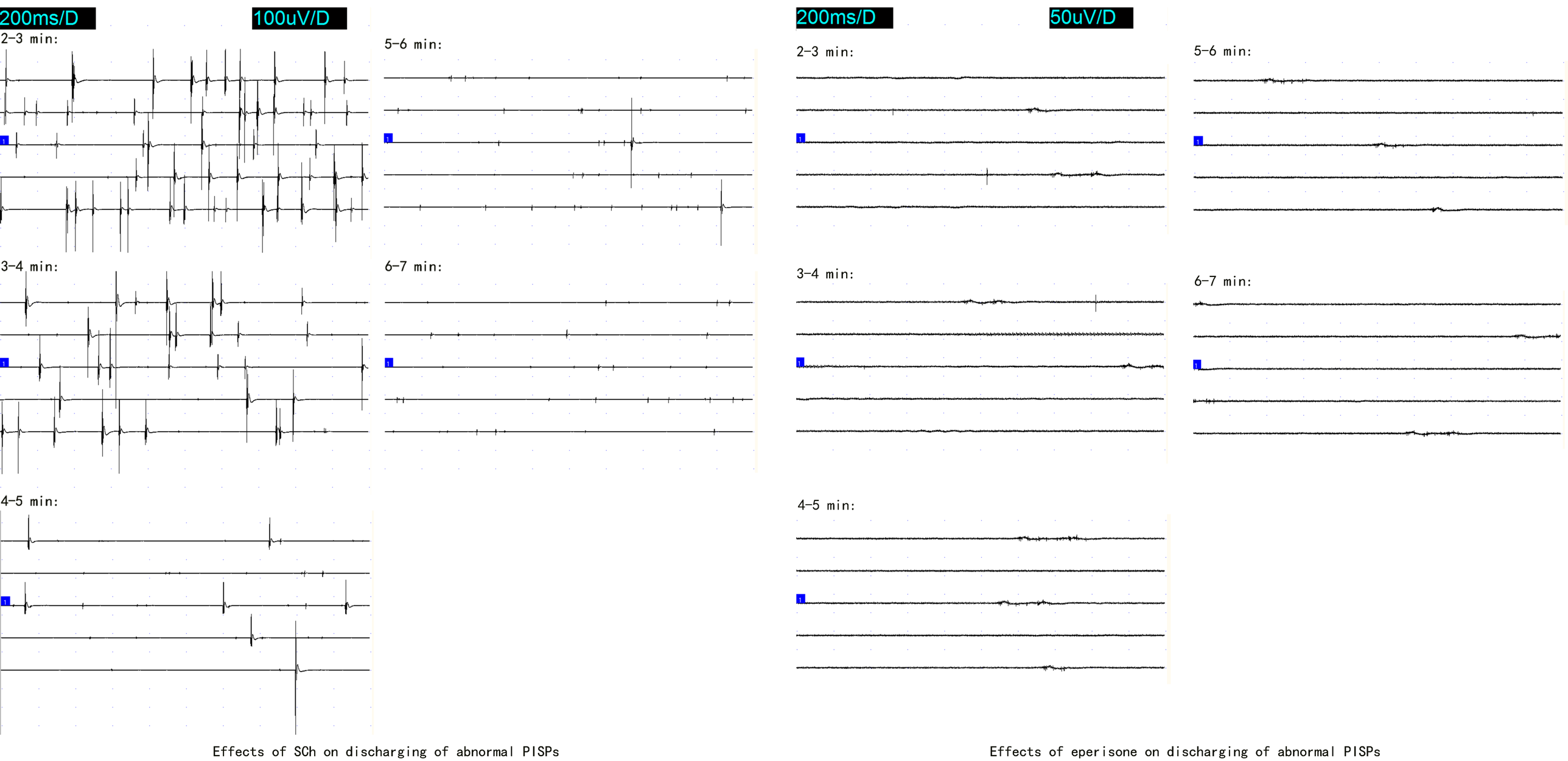

Supplement: Supplementary file 2 [file Image4.TIF]

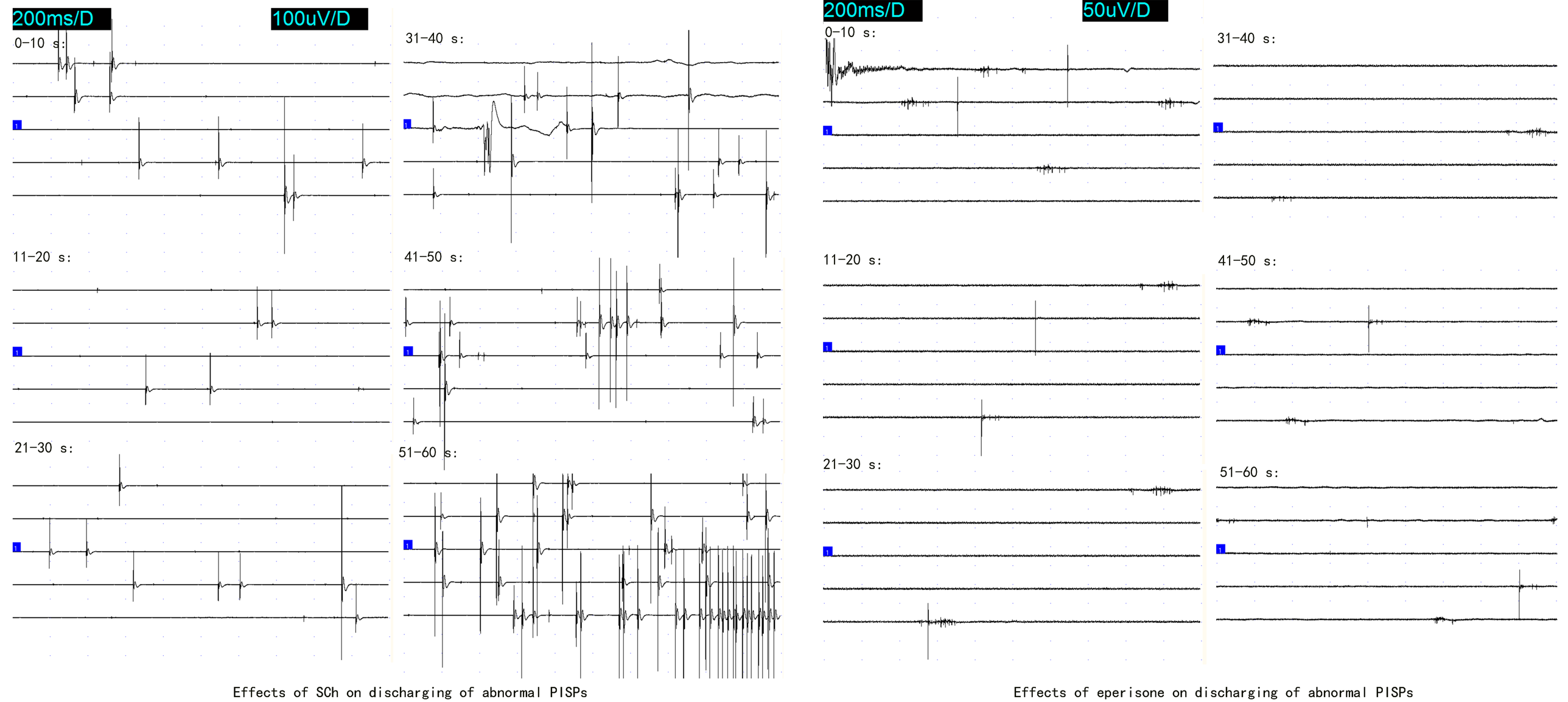

Supplement: Supplementary file 3 [file Image2.TIF]

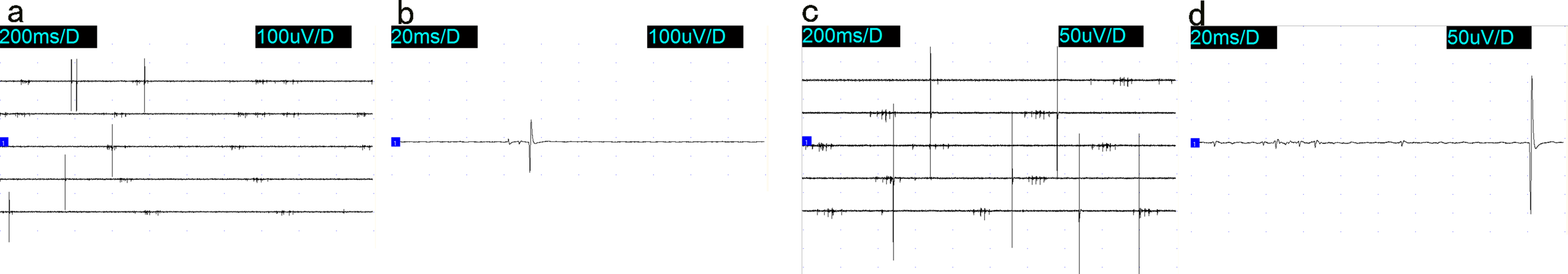

Supplement: Supplementary file 4 [file Image1.TIF]
